# A Single-Antenna Method and Post-Processing Strategy for Radar Cross-Section Measurements at Near-Field Ranges

**DOI:** 10.3390/s22197453

**Published:** 2022-09-30

**Authors:** Ilie Valentin Mihai, Andreea Constantin, Stefania Bucuci, Razvan D. Tamas

**Affiliations:** 1 Department of Electronics and Telecommunications, Constanta Maritime University, 900663 Constanta, Romania; 2 Doctoral School of Electronics, Telecommunications and Information Technology, University Politehnica of Bucharest, 060042 Bucharest, Romania

**Keywords:** single-antenna technique, radar cross-section, near-field, real environment, horn antenna

## Abstract

The single-antenna technique proposed in this paper was developed for measuring the radar cross-section at near-field distances in a real environment, from reflection coefficient measurements on the antenna. The near-field radar cross-section is corrected with an analytical factor calculated as a ratio between the radar cross-section computed at far-field and near-field. The analytical correction factor takes into account the effects of the diffraction at the edges of the target at incidence angles higher than 20°. An improved, distance averaging technique is proposed to reduce the multipath propagation effects. A time-gating procedure is additionally used in order to better isolate the reflection from the target and to remove the real environment contributions. The method was successfully tested on a rectangular metallic plate as a target over a wide frequency band, at normal and oblique incidence angles; however, it might also work for arbitrarily shaped targets, because they can actually be divided into small rectangular patches.

## 1. Introduction

Generally, the radar cross-section (RCS) is measured in anechoic chambers (AC) at near-field distances. Large targets, such as aircraft, ships, or terrestrial vehicles require large anechoic chambers and, therefore, the cost of such a measurement would be prohibitive. In this case, the RCS measurements in a real environment at near-field distances could be a strong alternative to the anechoic chamber measurements [1,2,3].

The RCS evaluation is usually performed by measuring the scattering parameters in monostatic, quasi-monostatic, or complex bistatic configurations with two antennas often exhibiting a strong mutual coupling, and therefore not suitable for measurements at oblique incidence angles [4]. In order to overcome these shortcomings, methods for measuring the RCS with a single-antenna setup in anechoic or reverberation chambers were recently developed [5,6,7,8]. In [5], the RCS of a metallic object is evaluated with a single antenna through measurements performed within a diffuse-field environment produced in a reverberation chamber (RC). The method presented in [6] consists in measuring the $S_{11}$ parameter of an antenna located in an RC in order to measure the RCS of a rectangular target. In [7], the authors introduce a measurement method for RCS estimation with a single transmitting and receiving antenna by investigating the backscattered field produced by a target. Compared to the classical aforementioned measuring methods in reverberation chambers, measuring the far-field RCS with a single antenna at near-field distances in a real environment requires a simpler and more compact measurement setup.

A representative approach to evaluate the RCS at near-field distances consists of performing near-field to far-field transformations by using mathematical relations between the scattered field and the current density on the target [9,10,11,12,13,14,15,16,17]. An algorithm derived from an optical model for estimating the surface currents induced on a scatterer by the incident field is proposed in [9], by using a special weighted version of the Fourier transform in order to calculate the near scattered field originating from the surface currents. An integral formulation of the mutual coupling and scattering, based on spherical angular functions is presented in [12]; an approximate deconvolution technique for nonplanar surfaces is proposed as well. The algorithm proposed in [14] corrects for all deviations of the actual, illuminating field from the assumption of a plane wave. Complex correction coefficients for these deviations are calculated for a region of a size equal to or greater than the dimensions of the test target. Convolving the correction coefficients with the RCS pattern of the target removes from the measurements the errors due to the non-plane wave illumination.

All aforementioned methods have a rather high computational efficiency but a quite poor extrapolation accuracy due to the surface current approximation.

In this paper, we propose a single-antenna method for measuring the RCS of a target in the near-field and in a real, multipath environment. The multipath propagation effects are reduced by applying an improved distance averaging technique combined with a time-gating procedure following a specific strategy for an optimal choice of the post-processing technique. A correction factor made it possible to validate our method at near-field distances and at oblique incidence angles where the diffraction effects are strong. The diffraction effects are calculated by defining equivalent edge currents on the side of the target and by using the diffraction coefficients developed by Kouyoumjian in [18]. The measurements are performed in a wide frequency range with a single horn antenna.

## 2. Theory

### 2.1. Radar Cross-Section Formulation

The RCS in the far-field zone is defined as

(1) $$\sigma =\lim_{r\to \infty }4\pi r^{2}\frac{|H_{s}{|}^{2}}{|H_{i}{|}^{2}}=\underset{=\sigma_{PO}}{\underset{︸}{\lim_{r\to \infty }4\pi r^{2}\frac{|H_{r}{|}^{2}}{|H_{i}{|}^{2}}}}+\underset{=\sigma_{Diff}}{\underset{︸}{\lim_{r\to \infty }4\pi r^{2}\frac{|H_{d}{|}^{2}}{|H_{i}{|}^{2}}}},$$

where *r* is the radar-to-target distance, and $H_{i}$ and $H_{s}$ are the incident and scattered magnetic field strength at the radar, respectively. It should be noted that the scattered magnetic field includes the reflected magnetic field strength $H_{r}$ and diffracted magnetic field strength $H_{d}$ at the radar, $\sigma_{PO}$ is the RCS based on the physical optics approximation, and $\sigma_{Diff}$ is the term corresponding to the diffraction effects [19].

**Figure 1:**
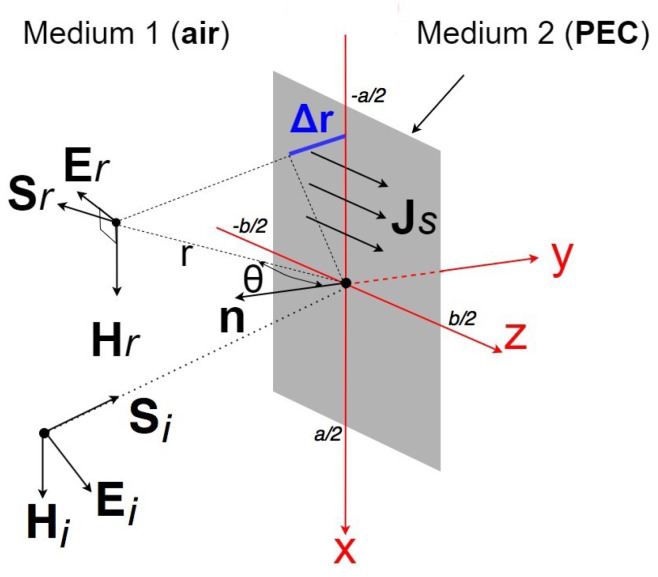
Magnetic field radiated by a rectangular plate.

The magnetic field reflected by a rectangular plate of a size *a* by *b* (Figure 1) can be expressed as follows:

(2) $$H_{r}=\int_{-\frac{b}{2}}^{\frac{b}{2}}\int_{-\frac{a}{2}}^{\frac{a}{2}}j\frac{kJ_{S}\cos\theta }{4\pi r}\exp\left[-jk\left(r+\Delta r\right)\right]dx\prime dz\prime ,$$

where *k* is the wavenumber, θ is the incidence angle, $J_{S}$ is the surface current density, and Δr is the path length difference.

**Figure 2:**
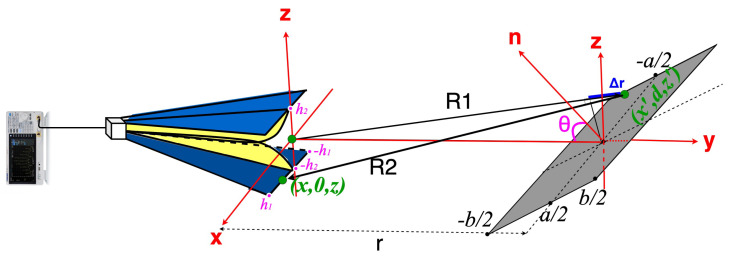
Radar type setup with a single antenna.

By employing Equation (2) in (1), in a radar configuration with a single antenna (Figure 2), $\sigma_{PO}$ for a rectangular target can be expressed as follows:

(3) $$\sigma_{PO}=\frac{4\pi \cos^{2}\theta }{\lambda^{2}}{\left|\frac{1}{\left(4h_{1}h_{2}\right)}\int_{-h_{1}}^{h_{1}}\int_{-h_{2}}^{h_{2}}\int_{-\frac{b}{2}}^{\frac{b}{2}}\int_{-\frac{a}{2}}^{\frac{a}{2}}\exp\left(-jk\Delta r\right)dx^{\prime }dz^{\prime }dxdz\right|}^{2}.$$

In (3), λ is the wavelength and an average over the antenna aperture is performed in order to further reduce the number of integrals and, therefore, the computing time for the near-field zone.

When the distance *r* is in the near-field range, for most practical antenna sizes and measuring ranges, Δr can be approximated as

(4) $$\begin{array}{c} \Delta r=2\left(R_{1}-r\right)=\frac{{\left(x^{\prime }-x\right)}^{2}+{\left(z^{\prime }-z\right)}^{2}}{r+z^{\prime }\sin\theta }+2z^{\prime }\sin\theta . \end{array}$$

By using (4) in (3), the RCS at near-field ranges $\sigma_{PO_{nf}}$ can be easily computed.

At far-field distances, the RCS of a rectangular target of sizes *a* by *b* becomes

(5) $$\begin{array}{cc} \sigma_{PO_{ff}} & =\frac{4\pi \cos^{2}\theta }{\lambda^{2}}{\left|\int_{-\frac{b}{2}}^{\frac{b}{2}}\int_{-\frac{a}{2}}^{\frac{a}{2}}\exp\left(-2jkz^{\prime }\sin\theta \right)dx^{\prime }dz^{\prime }\right|}^{2}\\ \; & =\frac{4\pi a^{2}b^{2}\cos^{2}\theta }{\lambda^{2}}{\left[\sin c(kb\sin\theta )\right]}^{2}. \end{array}$$

The diffraction term in (1) can be computed by using the equivalent currents technique [20].

The electric field diffracted by a wedge when the incident field is of a soft-type polarization [18] is

(6) $$\begin{array}{c} E_{d}^{z}=E_{i}D_{s}\frac{1}{\sqrt{2r}}\exp\left(-jkr\right), \end{array}$$

where $D_{s}$ is the diffraction coefficient at a soft-type polarization.

The magnetic field diffracted by a wedge when the incident field is of a hard-type polarization is

(7) $$\begin{array}{c} H_{d}^{x}=H_{i}D_{h}\frac{1}{\sqrt{2r}}\exp\left(-jkr\right), \end{array}$$

where $D_{h}$ is the diffraction coefficient at a hard-type polarization.

When assessing the diffraction on a plate edge along the *z* axis, the far magnetic field radiated by the equivalent electric line source of a length *b* can be written as

(8) $$\begin{array}{cc} H_{ds}= & j\frac{k\cos\theta \exp(-jkr)}{4\pi r}\int_{-\frac{b}{2}}^{\frac{b}{2}}I_{z}\exp\left(-jk\Delta r\right)dz^{\prime }, \end{array}$$

where $I_{z}$ is the equivalent electric current.

A similar form can be derived for the magnetic field originating from a magnetic finite line source of a length *a* along the *x*-axis

(9) $$\begin{array}{cc} H_{dh}= & j\frac{k\exp(-jkr)}{4\pi Z_{0}r}\int_{-\frac{a}{2}}^{\frac{a}{2}}M_{x}\exp\left(-jk\Delta r\right)dx^{\prime }, \end{array}$$

where $M_{x}$ is the equivalent magnetic current.

Finally, the diffracted-to-incident magnetic field ratio can be expressed as follows:

(10) $$\begin{array}{c} \frac{H_{d}}{H_{i}}=\frac{2(H_{dh}+H_{ds})}{H_{i}}. \end{array}$$

### 2.2. Near to Far-Field Correction Factor and Multipath Effect Reduction

By using $\sigma_{PO}$, $\sigma_{PO_{nf}}$ and $\frac{H_{d}}{H_{i}}$, the ratio computed at near-field and far-field ranges as in (10), one can further define a correction factor *F* between near- and far-field zones as follows:

(11) $$F=\frac{\sigma_{PO_{nf}}+\sigma_{Diff_{nf}}}{\sigma_{PO_{ff}}+\sigma_{Diff_{ff}}},$$

where $\sigma_{Diff_{nf}}$ and $\sigma_{Diff_{ff}}$ are the RCS terms corresponding to the diffraction effects computed at near-field and far-field ranges, respectively.

In a single-antenna radar configuration, the far-field zone radar equation yields [21]

(12) $$\sigma_{ff}=\frac{P_{r}{\left(4\pi \right)}^{3}r^{4}}{P_{t}G^{2}\lambda^{2}},$$

where $P_{r}$ is the received power, $P_{t}$ the transmitted power and $\sigma_{ff}$ the RCS measured at far-field distances. The ratio $\frac{P_{r}}{P_{t}}$ is extracted from the $S_{11}$ parameter of the antenna.

When measuring the RCS in a real site, in order to reduce the environment impact, we subtracted the reflection coefficient measured without the target ($S_{11}^{no}$) from reflection coefficients measured with the target placed at distances in the near-field range ($S_{11,n}$). Let $S_{11}^{refl}$ be the contribution of the target scattered field to the reflection coefficient at the antenna input. By using the correction factor analytically computed as in (11), one can find the far-field RCS from measurements at near-field distances:

(13) $$\begin{array}{cc} \sigma_{ff}= & \frac{{\left(4\pi \right)}^{3}|S_{11}^{refl}{|}^{2}d_{0}^{4}}{FG^{2}\lambda^{2}}. \end{array}$$

In (13), $d_{0}$ is a reference distance of 1 m and a normalized, distance-averaged reflection coefficient $S_{11}^{total}$ was used in order to compensate the effects of the multipath propagation for measurements in a real environment

(14) $$S_{11}^{total}=\frac{1}{N}\sum_{n=1}^{N}[(\frac{d_{n}}{d_{0}}{)}^{2}\left(S_{11,n}-S_{11}^{no}\right)\exp\left(2jkd_{n}\right)],$$

where $d_{n}$ are a set of near-field distances between antenna and target.

**Figure 3:**
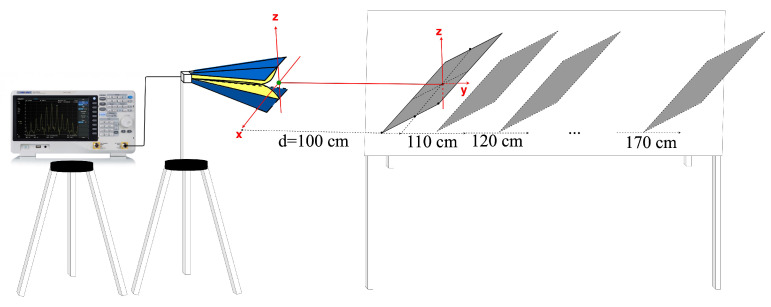
Proposed distance averaging technique.

The distance-averaging technique proposed in (14) is a new version of the technique originally presented in [22], as the average is computed on a set of input reflection coefficients measured at distances in the near-field range (Figure 3).

## 3. Method Validation by Simulations and Measurements

In order to validate the method, simulations and measurements were performed at frequencies between 3 and 11 GHz. We choose as a target a rectangular, metallic plate of 22 cm by 35 cm, and we used a horn antenna with an aperture size of 20 cm by 20 cm.

**Figure 4:**
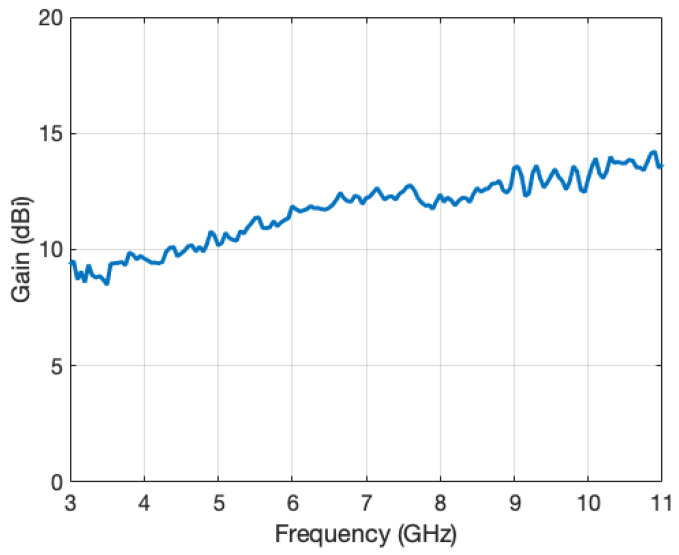
Antenna gain as a function of frequency in the main direction of radiation.

**Figure 5:**
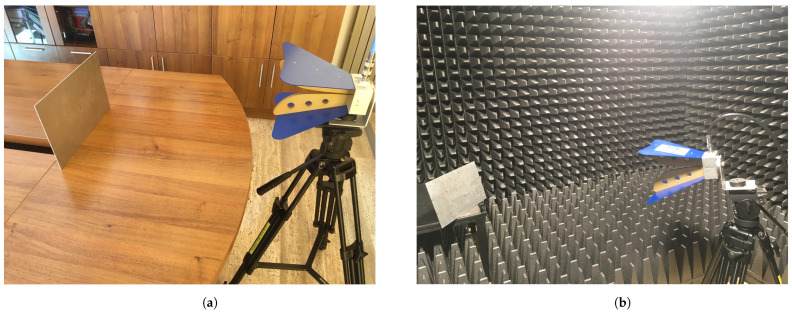
Measurement configuration in a real environment (**a**) and in an anechoic chamber (AC) (**b**).

The antenna was a standard ridged horn (model PowerLog 70180 by Aaronia), operating in the frequency range from 700 MHz to 18 GHz. The gain variation in the main direction of radiation over the frequency range of interest is given in Figure 4. A vector network analyzer (Anritsu MS2038C) was used for measuring the reflection coefficient of the antenna in a real, multipath environment (Figure 5a), and in an anechoic chamber, respectively (Figure 5b).

Most radars for discovering targets hidden by vegetation use low frequencies, typically in the VHF and UHF bands. Outdoor tests in a real scenario would require long ranges and high power levels. We therefore tested our technique by using a scale-reduction approach. As a multipath environment we chose an indoor site i.e., a regular office room. In that case, distances and typical target sizes can be reduced by a factor of ten, whereas the frequencies are multiplied by the same factor. The power level delivered by the VNA internal generator is enough for a good signal-to-noise ratio.

**Figure 6:**
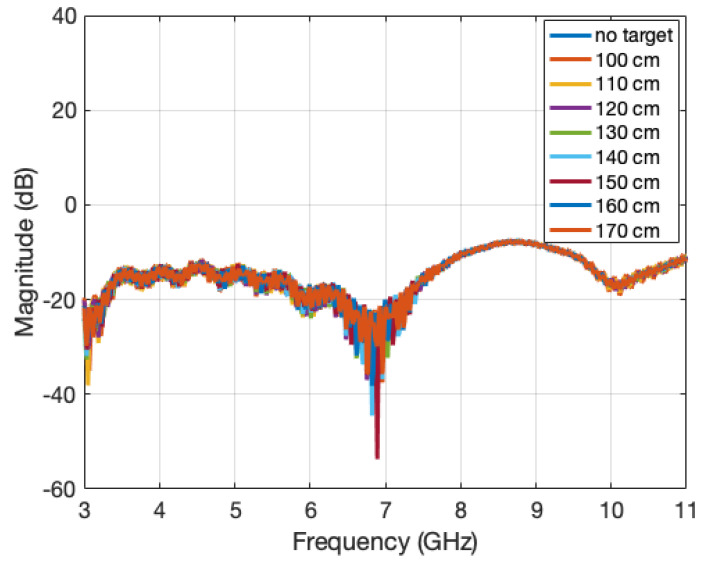
Input reflection coefficient of the horn antenna measured at eight distances far away from the target.

The $S_{11}$ parameter was measured for eight ranges equally spaced between 100 and 170 cm, all of them in the near-field zone (Figure 6).

When measuring the reflection coefficients on a planar target in a real environment, as the angle of incidence increases, the magnitude of the scattered field from the target becomes comparable to the that of the scattered field from other objects. In addition, the application of the distance averaging technique (DA) would reduce the effects of the diffraction not only on the environing objects, but also on the target edges [22]. In that case, a subtraction of the reflection coefficient measured without target from the reflection coefficient measured with the target might not yield reliable results. In order to validate our method at oblique incidence angles, we therefore combined our technique with a traditional method for nonanechoic sites—that is, the time-gating method (TG). That is, we set the distance between antennas between 100 and 170 cm and a time gating on the inverse Fourier transform of the reflection parameter was also performed at each measuring position. The width of the time gate took into account the propagation delay, the delay through the measuring antenna, and the estimated length of the time-domain response of the system. It was finally set to 12 ns. The time gating was performed as part of the post-processing, and the window shape was rectangular.

**Figure 7:**
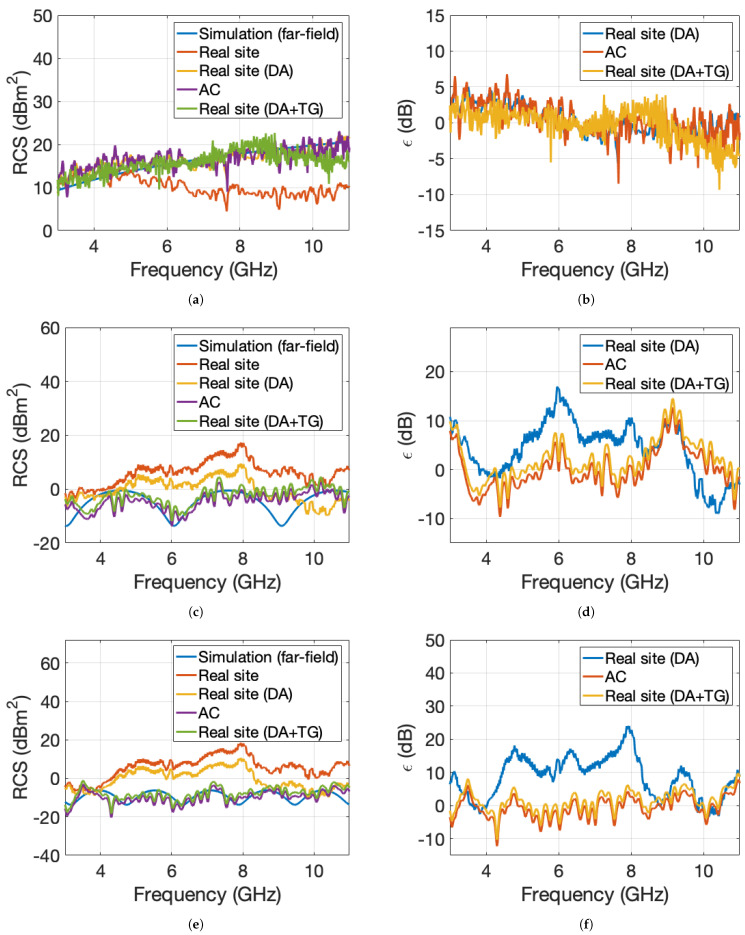
Comparison between simulated far-field RCS, RCS measured at near-field distances, RCS measured at near-field distances corrected with F (in an AC or in a real site, after applying DA or a combination of DA and TG), at θ = 0° (**a**), θ = 15° (**c**) θ = 25° (**e**). Absolute error between the RCS measured at near-field distances corrected with *F* (in an AC or in a real site, after applying DA or a combination DA+TG), and the far-field RCS simulations at θ = 0° (**b**), θ = 15° (**d**) θ = 25° (**f**).

**Figure 8:**
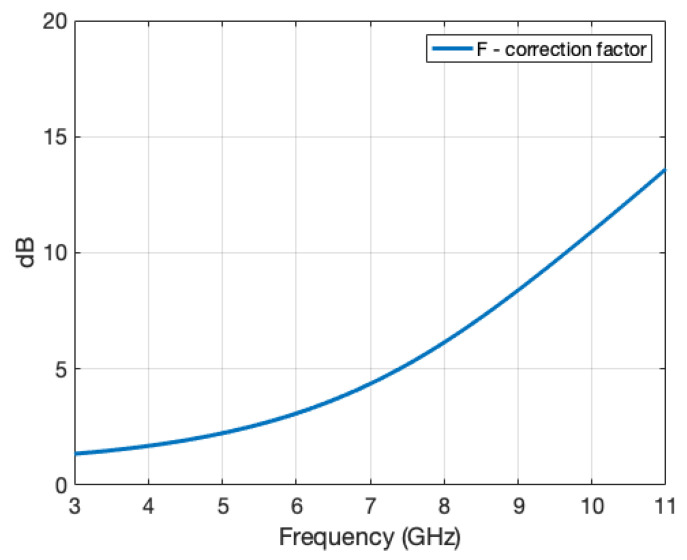
The F correction factor.

Figure 7a,c,e shows the comparison between simulated far-field RCS, RCS measured at near-field distances, and RCS measured at near-field distances corrected with *F* at θ = 0°, 15°, and 25°. At such low incidence angles, the correction factor mostly varies with frequency, and less with the distance to the target edges. Figure 8 shows the correction factor as a function of frequency. The measurements were performed in an AC and in a real site, by applying DA or a combination DA+TG.

The absolute error between the RCS measured at near-field distances corrected with the *F* factor (in an AC or in a real site, after applying DA or a combination DA+TG), and the far-field simulations is displayed in Figure 7b,d,f.

In order to assess the accuracy of the results regarding the field zone correction and the techniques proposed to reduce the multipath propagation effects, a relative mean error (RME) was calculated. The RME in (15) was calculated for the RCS measured at near-field distances corrected with *F* (in an AC or in a real site, after applying DA or a combination DA+TG), by taking as reference the simulations of the RCS in the far-field zone (Table 1):

(15) $$\epsilon_{RME}=\frac{1}{N}\left|\sum_{i=1}^{N}\frac{\sigma_{DA/AC/DA+TG}\left(i\right)-\sigma_{simulation}\left(i\right)}{\sigma_{simulation}\left(i\right)}\right|,$$

where $\sigma_{simulation}\left(i\right)$ is the far-field RCS simulated at the *i*-th frequency in the range.

As expected, the RCS obtained after the utilization of the DA at oblique incidence is very far from far-field simulated RCS. Instead, by utilizing a combination between the DA and TG, the absolute error between the measurements and the far-field RCS rarely exceeds 5 dB over the entire 3–11 GHz band. Thus, when measuring the RCS at near-field distances an adaptive choice between the post-processing methods (DA or DA+TG) is needed, depending on the frequency and angle of incidence.

## 4. Conclusions

We proposed a novel single-antenna method and strategy of post-processing for RCS measuring in the near-field-zone. The simulated, far-field RCS is in good agreement with the RCS measured in the near-field region at normal incidence and in a real environment, by applying DA. Conversely, at oblique incidence a combination between DA and TG as post-processing techniques would lead to more accurate results than the DA alone. In any case, the relative mean error over the frequency range of interest, compared to the far-field simulation is less than 15%. The technique proposed in this paper may reduce the cost of the RCS measurements by using either smaller anechoic chambers, or by performing real site measurement. Future research will focus on optimizing the method to measure the RCS of simplified models consisting of rectangular patches and slots; the far-field RCS of such a model can further be evaluated analytically for comparison purposes. In order to extend the method to dielectric targets, the $\frac{H_{r}+H_{d}}{H_{i}}$ ratio will be analytically derived by taking into account the dielectric permittivity. Measurements on full-scale targets in a real outdoor environment, and at real operating frequencies (in the VHF and UHF bands) will be performed in a future work as an additional test of our method accuracy.

## Figures and Tables

**Table 1:** Relative mean error ($\epsilon_{RME}$) calculated for the RCS measured at near-field distances corrected with *F* (in an AC or in a real site, after applying DA or a combination DA+TG), by taking as reference the RCS simulated at far-field.

	DA	AC	DA+TG
0°	0.04	0.05	0.00
15°	0.95	0.11	0.15
25°	0.89	0.10	0.12

## Data Availability

The data that support the findings of this study may be available from the corresponding author upon reasonable request.
